# Visuoperceptual Impairment in Children with NF1: From Early Visual Processing to Procedural Strategies

**DOI:** 10.1155/2019/7146168

**Published:** 2019-01-13

**Authors:** Sara Bulgheroni, Matilde Taddei, Veronica Saletti, Silvia Esposito, Roberto Micheli, Daria Riva

**Affiliations:** ^1^Developmental Neurology Division, Fondazione IRCCS Istituto Neurologico Carlo Besta, Via Celoria 11, 20133 Milan, Italy; ^2^Pediatric Neuropsychiatry, Spedali Civili of Brescia, Piazzale Spedali Civili 1, 25125 Brescia, Italy

## Abstract

Visual-spatial impairment has long been considered a hallmark feature of neurofibromatosis type 1 (NF1). No study investigating the cognitive and neuropsychological profile of NF1 used the Rey Complex Figure Test (RCFT) task as the primary measure of visual-perceptual abilities taking into consideration all functions involved including the strategic processing style. We compared 18 children with NF1, 17 siblings (S), and 18 typically developing children (TD) at intelligence scale and RCFT copy, recall, and recognition trials; we also evaluated the copy strategy as a measure of a visual-processing style. Children with NF1 had normal total IQ, with cognitive weaknesses in the perceptual organization and working memory in line with the existing literature. At the RCFT copy, immediate and delay recall scores are significantly lower in NF1 than S and TD, while recognition is in the normal range in all groups. Copy style was poor and less efficient in children with NF1 and correlated to copy and recall ability, but the effect of the group in the RCFT copy and recall remained significantly controlling for strategic approach. The present study confirms visuospatial impairment in children with NF1, due to a deficit in perceptual analysis of shape and their spatial features, in visuomotor integration efficiency and strategies, in recall memory, while recognition memory is preserved. A more configural/holistic style may facilitate both the visual-perceptual and visuomotor ability and the recall process. Visuoperceptual impairment in NF1 seems to be a unified process from early visual processing to higher order functions (planning, strategy, and executive functioning).

## 1. Introduction

Neurofibromatosis type 1 (NF1) is an autosomal dominant genetic disorder with an incidence of approximately 1 in 2700 individuals [1]. Individuals with NF1, in addition to clinical characteristics (café-au-lait spots, multiple neurofibromas, and bone deformities), also have a high incidence of macrocephaly, optic gliomas, and T2-weighted hyperintensities (T2H) on brain MRI. Deficits in language and cognitive domains have been extensively reported in people with NF1 [2, 3]. In particular, visual-spatial impairment has long been considered a hallmark feature of the disorder, especially on the Judgment of Line Orientation Test [4], while evidence pertaining to other areas of behaviour, such as executive and motor functioning, verbal memory, and various linguistic skills, remains inconsistent [3]. These cognitive and behavioural impairments have a substantial impact on the quality of life and are a major concern for parents and teachers [5].

Moreover, although still little is known about the neural substrates underlying executive control and visuospatial abilities deficits, there is some evidence suggesting that documented brain structural and functional abnormalities are related to the NF1 behavioural profile [6–8].

In the visuospatial domain, the most common deficits involve visual-spatial analysis [9], spatial learning and memory [10], visual-motor integration skills [11–14], and perceptual organization [15].

Several studies investigated visuospatial skills, using a variety of measures that focus on perception of angulation (e.g., the Judgment of Line Orientation Test), visual organization (e.g., the Hooper Visual Organization Test), and object recognition (e.g., Benton Visual Form Discrimination Test and the Birmingham Object Recognition Battery) [14, 16–19].

Children with NF1 have been found to perform significantly more poorly on visuoperceptual tasks than siblings [2, 10, 13, 19–25] as well as scoring below average for their age [26, 27]. However, some studies have reported conflicting results, e.g., showing larger deficit in visuoperceptual tasks than in tasks requiring visuomotor integration [28, 29].

In relation to conflicting results in the literature, it is important to acknowledge that very often the sample size was fairly small [28, 29] and only a small proportion of children with NF1 performed 2 SD or more below the mean for their age [3].

Studies on children with NF1 focusing on memory have reported inconsistent results, especially for verbal memory, with a slightly more consistent finding in visual memory for a complex figure, i.e., the delayed recall condition of the RCFT, in which children with NF1 tend to perform poorly [20, 21, 26, 29].

These numerous conflicting results are likely due to several factors such as limitation in study design, use of different testing measures, and biased clinical populations (i.e., inclusion of patient with brain tumours or other brain pathologies and associated psychiatric conditions) but also reflect the range of cognitive and neuropsychological phenotypes of NF1 population.

A recent study by Van Eylen and collaborators [30] investigated the role of possible confounding factors in visuoperceptual impairments in children with NF1. The authors concluded that the reduced performance on a visual-integration task and the more detail-oriented processing style in subjects with NF1 appeared to result from confounding executive functions impairment, while the co-occurring autism spectrum disorder symptoms and lower verbal IQ did not substantially impact the visuoperceptual performance.

The RCFT is a widely used neuropsychological test that requires the analysis and reproduction of an unfamiliar, nonmeaningful figure and is used to assess visual-perceptual organization and memory processes [31, 32]. RCFT has been partially employed in a number of studies investigating cognitive and neuropsychological profile of NF1, alone or included in neuropsychological batteries [3, 9, 26, 30].

To our knowledge, no study used the complete RCFT task as a primary measure of visual-perceptual abilities, taking into consideration all functions involved, i.e., perceptual analysis, integration and processing-strategic style, and visual-spatial memory.

The objective of the present study was to better examine the visuoperceptual impairment in NF1 in developmental age, providing new data about the specificity, magnitude, and frequency of this deficit. For this purpose, we compared patients to their unaffected sibling and children of typical development at the RCFT considering all functions involved, including processing-strategic style. We hypothesized different cognitive and neuropsychological profiles in the NF1 compared to siblings and normal peers, in the direction of a reduced visuospatial accuracy and less-efficient visual-processing style.

To overcome the limitations of the previous studies, we employed a strict selection of the sample (elimination of patients with brain tumours, intellectual disability, and neurological conditions like epilepsy) controlling the principal confounding factors. Moreover, the use of RCFT permits the examiner to assess different domains of visual processing without the need to include a wide variety of different psychometric instruments, allowing the study to remain in a defined theoretical framework [3].

## 2. Methods

### 2.1. Participants

This case-control study is part of a research project carried out between 2009 and 2012. Participants were recruited from patients attending two clinical settings specialized in the care of children with NF1 located in Brescia and Milan, Italy.

We evaluated 18 children with NF1 (NF1), 11 males and 7 females aged between 6 years 11 months and 16 years and 3 months (mean: 10 years and 2 months, SD: 2 years and 6 months); 17 unaffected siblings (S), 9 males and 8 females aged between 6 years and 11 months and 21 years and 4 months (mean: 12 years and 3 months, SD: 4 years); and 18 typically developing controls (TD), 11 males and 7 females aged between 6 years and 9 months and 16 years and 4 months (mean: 10 years and 3 months, SD: 2 years and 11 months). The evaluation of unaffected siblings was included to increase the specificity of the study design. The TD and sibling groups allow to control for gender and age and for social and genetic background, respectively.

Clinical evaluation included neurological examination, brain MRI, genetic testing, ophthalmological examination including assessment of vision, refraction, biomicroscopy and fundoscopy, and dermatologic evaluation. Participants with the following findings were excluded from the study: brain tumours (including optic glioma), intellectual disability (full-scale IQ < 75), other developmental disorders including autism spectrum disorder, basic visual impairment (i.e., problems with visual acuity, visual field, and/or colour vision), associated diseases such as epilepsy, and hydrocephalus.

The presence of behavioural problems, including attention-deficit hyperactivity disorder- (ADHD-) like behaviours, was assessed by parent-completed questionnaires (Child Behavior Checklist 6-18 [33] and Conners Rating Scale-Revised [34]).

The study was approved by the institutional Ethics Committee, and written informed consent was obtained from the parents of all participants.

### 2.2. Neuropsychological Measures

Patients, unaffected siblings, and typically developing control subjects were assessed in a quiet room by examiners who had specific training on the child assessment.

#### 2.2.1. Intelligence

Intelligence was evaluated using the Wechsler Intelligence Scale for Children-III (WISC-III) [35, 36]. For each of the 53 participants, full-scale IQ and four factorial indices verbal comprehension (VC), perceptual organization (PO), freedom from distractibility (FFD), and processing speed (PS) were calculated.

#### 2.2.2. Visuoperceptual Processing and Memory

Visual, spatial, constructional, and strategic abilities and visual memory were evaluated using the RCFT. Detailed RCFT Administration procedures are provided in the Supplementary Materials. We chose to administer the 1995 version of J. E. Meyers and K. R. Meyers [37] since it includes supplemental norms for children and adolescents [38] and a recognition trial, in which the subject is required to encircle each component figure that belongs to the whole design just drawn. This trial has been found to be age and education free and clinically useful when combined with the RCFT recall measures, providing incremental discriminatory power in distinguishing brain-injured patients, psychiatric patients, and healthy normal subjects compared to using only the recall variables [39]. Moreover, recognition memory may be relatively intact even when recall is poor or when the child is hesitant to guess. The following RCFT measures were considered outcome variables: copy, immediate recall, delay recall, and recognition. One senior neuropsychologist (BS) administered the RCFT test, and one junior neuropsychologist (TM), specifically trained and blind about the groups, performed the evaluation and scoring of the drawing reproductions.

The quality of the copying process was evaluated for accuracy (form reproduction quality of each element) and placement (correct location of each element in relation to the general gestalt and to the other elements). Each of the 18 scoring elements of the complex figure stimulus was evaluated on a four-point scale (0-0.5-1-2), according to the instructions of the manual. Moreover, we separately considered the accuracy and placement scores, evaluating each element on a three-point scale (0-0.5-1).

Finally, to investigate *how* the subject approached the task, we also evaluated the copy strategy as a measure of visual processing style. As the participants had to copy the rey figure without explicit instructions on how to do so, the drawing style was rated by a 4-point scale graded as follows: 1—detection of the armature; 2—details of armature; 3—juxtaposition of contiguous details; and 4—details on a confused ground with little structure in which the global model is poorly recognizable.

### 2.3. Statistical Analysis

Statistical analyses were employed by SPSS Statistics 20 software [40].

The IQ age-scaled subscores were normally distributed with a mean of 10 and a SD of 3. The sum of age-scaled scores was converted to an overall standard score ranging from 49 to 146, with a mean of 100 and a standard deviation of 15.

The RCFT raw scores were converted to *z*-scores with a mean of 0 and a standard deviation of 1.0.

Repeated measure analysis of variance (ANOVA) was carried out to compare the *z*-scores in copy, immediate, and delay recall, and the paired *t*-test was performed to investigate the difference between delayed recall and recognition within groups.

One-way ANOVA was used to compare the standard scores on WISC-III and RCFT for the three groups (NF1, S, and TD), and the post hoc Bonferroni test was carried out to investigate between group differences. Moreover, the influence of the copy strategy as a potential confounding factor was tested by adding the strategy measure as a covariate to the model (ANCOVA).

Furthermore, we performed ANOVA to separately analyse group differences on accuracy and placement raw scores.

The copy strategy measure was a nominal variable; the *χ* ^2^ and Fisher's exact probability test were carried out to compare the copy strategy among the three groups, and the adjusted Pearson residual analysis (or corrected standardized residuals) was performed to identify observed frequencies significantly higher or lower than expected frequencies.

The *χ* ^2^ and the Fisher's exact probability test were also carried out to compare the frequencies of subjects with different performance levels in the three groups (≥-1 SD normal, <-1 SD borderline, and ≤-2 SD impaired).

Spearman's correlation analyses were conducted to test the following association: RCFT copy, recall, and recognition scores with strategic style and RCFT copy score and strategies with scores at attention problem scales.

All the statistical analyses were two-tailed, and *p* values of 0.05 or lower were considered significant.

## 3. Results

### 3.1. Demographic and Behavioural Characteristics of the Sample

The three groups NF1, S, and TD were comparable for sex (*χ* ^2^ = 0.315, *p* = 0.940 by Fisher's exact test) and age (*F* = 2.253, *p* = 0.116).

For what concerns behavioural and emotional features, 2 children with NF1, 1 sibling, and 2 TD controls scored above clinical threshold (≥70) on affective or anxiety DSM-oriented CBCL scales. Moreover, 8 out of 18 NF1 patients (44%) scored over the significant cut-off at attention problem scales (Conners' ADHD index *T*-score ≥ 65 or CBCL ADHD DSM-oriented scale *T*-score ≥ 70), with a higher frequency than S and TD groups (1 subject in each group; *χ* ^2^ = 10.001, *p* = 0.004 by Fisher's exact test).

### 3.2. Neuropsychological Measures and Group Comparison

#### 3.2.1. Intelligence

Table 1 shows the mean, standard deviation, and group comparison on the WISC-III Intelligence test. The NF1 group had mean full-scale IQ within the normal range but significantly lower than both S and TD. Children with NF1 performed significantly lower than both control groups at perceptual organization (PO) and freedom from distractibility (FFD) indices, while no significant differences were found across the three groups on verbal comprehension (VC) and processing speed (PS), even if inferior than S and TD.

#### 3.2.2. RCFT (Copy, Recall, and Recognition)

Repeated measure ANOVA revealed no significant differences among copy, immediate, and delay recall mean *z*-scores within each of the three groups (NF1: *F* = 1.162, *p* = 0.338; S: *F* = 0.174, *p* = 0.842; and TD: *F* = 1.157, *p* = 0.339). The paired *t*-tests showed lower delay recall mean *z*-score compared to the recognition score only in the children with NF1 (*t* = −6.057, *p* ≤ 0.001).

Table 2 shows the mean, standard deviation, and group comparison on the RCFT *z*-scores. The NF1 group showed mean scores 1.5 SD below age expectation in the copy, immediate recall, and delay recall trials. Group differences were significant, and post hoc Bonferroni comparisons confirmed that children with NF1 performed lower than both the S and TD groups. The mean scores in the recognition trial showed no significant differences between groups.

Analyses considering separately the accuracy and placement scores revealed no difference in the accuracy scores, while the placement scores were significantly lower in NF1 only compared to TD peers (see Table 3).

#### 3.2.3. RCFT Performance Distribution among the Three Groups

Table 4 shows the distribution of the RCFT performance divided in three categories (*average*: *z*-score ≥-1 SD, *borderline*: *z*-score <-1 SD, and *clinical*: *z*-score <-2 SD) among the three groups. For all the RCFT measures (copy, immediate recall and delay recall), the *χ* ^2^ revealed significantly higher frequencies of clinically relevant, poor scores in the NF1 than in the S or TD groups. This result is in accordance with one-way ANOVA results.

#### 3.2.4. Copy Strategy and Visual Processing Style

The *χ* ^2^ revealed significant differences among the three groups with regard to the strategy of the copy used by participants (*χ* ^2^ = 13.867; *p* = 0.015 by Fisher's exact test). In the NF1 group, a significantly higher number of subjects carried out the worst strategy (details on a confused ground with little structure in which the global model is poorly recognizable), while in the TD group the use of details of armature strategy is significantly more frequent (see Table 5 for details).

**Figure 1:**
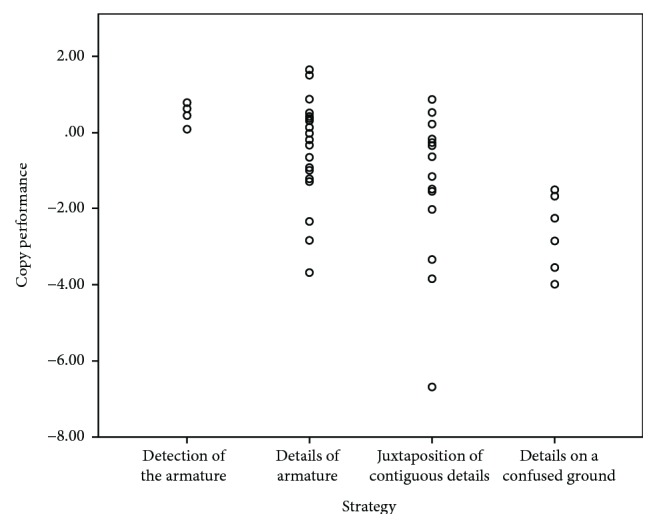
Correlation between RCFT performance and processing style. The figure shows the correlation plot between RCFT performance (*y*-axis) and the quality of copy strategy (*x*-axis).

**Figure 2:**
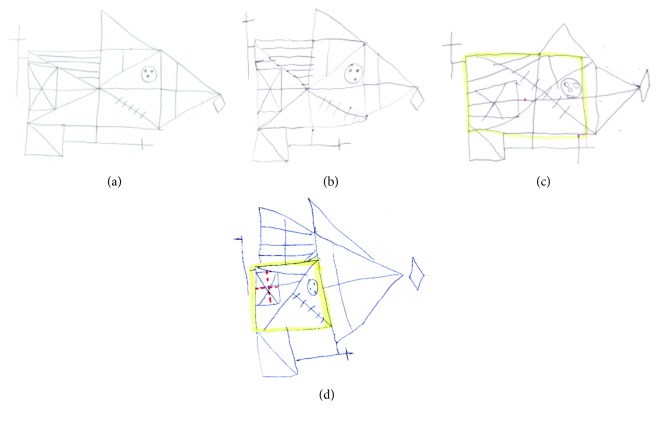
Copy performances and strategies. The figure shows different copy performances according to different procedural strategies: (2a) Female, 127 months, *z*-score copy = 0.78; detection of the armature. (2b) Male, 131 months, *z*-score copy = 0.50; details of the armature. (2c) Female, 139 months, *z*-score copy = −3.34, juxtaposition of contiguous details. (2d) Male, 116 months, *z*-score copy = −3.98; details on a confused ground.

Spearman's correlation analyses revealed a significant association between copy *z*-scores and the processing style quality score in the whole sample, as shown in Figure 1 (rho = −0.552, *p* ≤ 0.001). Different copy performances according to different strategies are presented by Figure 2. Significant correlations were also found between copy strategy and *z*-scores on the recall and recognition trials in the whole sample (immediate recall rho = −0.469, *p* ≤ 0.001; delay recall rho = −0.472, *p* ≤ 0.001; and recognition rho = −0.279, *p* = 0.045). When including the type of strategy as a covariate into the ANOVA model, the effect of the group on RCFT copy and recall measures remained significant (Table 2); post hoc tests revealed significant differences between NF1 and S, not between NF1 and TD.

#### 3.2.5. Correlation between Behavioural Problems and Copy Performances

No significant association by Spearman's correlations was found between *T*-scores at attention problem scale at the CBCL and CRS-R questionnaires and copy performances or strategic style, neither in the whole sample nor in each experimental group.

## 4. Discussion

To the best of our knowledge, this is the first study investigating intelligence and a broad range of visual-spatial abilities (shape and spatial relation perception, visual-motor integration, strategy, and memory) using RCFT, allowing the assessment of different visual-processing domains without the bias of using different measures [3].

The RCFT performances were evaluated in children with NF1 and compared to unaffected siblings and typically developing subjects.

Analyses of the intelligence scale profiles show normal full-scale IQ and factorial indices in NF1, despite that perceptual organization (PO) and freedom from distractibility (FFD) index scores are significantly lower than the controls.

The Wechsler scale results confirm previous evidences reporting that children with NF1 usually have an intellectual level within the normal range [3, 21, 24, 27, 28, 30]. In accordance with previous findings, our results indicate cognitive weaknesses in the perceptual organization and working memory, but comparable level to control groups in processing speed, verbal comprehension, and reasoning [3].

Group comparisons of the RCFT performances show that NF1 copy scores are below average (<−1.5 SD) and significantly lower than the S and TD groups. Both immediate and delayed recall scores are significantly lower in NF1, while recognition is in the normal range without group differences. In accordance with the ANOVA results, frequencies of clinically relevant scores in copy, immediate, and delay recall are significantly higher in NF1 than in S and TD.

From 16% to 83% of subjects, NF1 has normal performances, while between 0% and 44%, it has clinically relevant impaired performances (below 2 SD) on at least one RCFT measure (copy, recall, or recognition trial), confirming the wide heterogeneity of NF1 phenotype [2, 3].

As suggested by previous studies [17, 26, 41], RCFT result interpretation should account for the diversity of processes involved in the trial, i.e., the stimulus' perceptual and spatial analysis, the integration of different elements into a whole gestalt, the visual-motor integration processes, the executive planning, and the organization of strategies to copy and recall stimulus figure. Although neuropsychological tests and paradigms attempt to measure discreet aspects of cognition and behaviour, it is very difficult to have a single task not requiring the use of other skills in addition to the particular one that is measured. Constructs overlap by definition, for example, visuospatial, visuomotor, motor coordination skills, and executive function. Thus, it is very difficult to separate different domains unless artificially [3].

One or more of these processes may thus underlie a poor performance on the RCFT. However, the scoring system does not allow analysis of a subject's performance at different operating levels. To better investigate this issue, we analysed the accuracy of the copy both in terms of shape reproduction and patial relationships of each element, the quality of the strategy employed during the copy trial, and the relationship between strategy and the RCFT score for the copy trial.

Accuracy scores are not significantly different among groups, while the placement scores are less efficient in children with NF1, in particular when compared to TD children.

It is well known that visual-perceptual analysis and cognitive processing of visual and spatial stimuli occur in a complex network distributed throughout the brain, encompassing the primary visual cortex and a wide number of extrastriate cortical areas with modular functioning involved in higher-order visual processing [42–44]. According to the current prevailing conceptualization of visual processing (though under debate), i.e., the model of ventral and dorsal cortical pathways, visual processing is segregated and organized into two major subcortical streams to the midbrain, both arising from the primary visual cortex V1, dealing with the identification of the visual stimulus and the localization and orientation of the same stimulus, respectively [45, 46]. The double impairment showed by our NF1 subjects in reproduction of the shape but especially in the spatial placement of the stimulus' elements could be due to the malfunctioning of ventral and dorsal streams, respectively.

The neural explanation for the high-order cognitive deficits present in NF1 has been provided by fMRI studies investigating the early visual processing in children, adolescents and adults with NF1. Violante et al. [7] used two distinct stimuli, differing in contrast and spatial and temporal frequencies, designed to preferentially activate the magnocellular (dorsal stream) or parvocellular pathways (ventral stream); results showed that NF1 has deficient activation of the visual cortex, as compared to the control group, for both types of low-level visual stimulation preferentially involving dorsal and ventral streams, respectively. Accordingly, hypoactivation in the primary visual cortex has been reported in patients with NF1 during the Judgment of Line Orientation Test, known to be impaired in NF1 and associated to dorsal stream recruitment [28]. Neuroanatomical abnormalities (i.e., lower local gyrification) localized in the right cuneus and pericalcarine structures [47] and biological explanation related to reduced GABA levels in visual cortex in NF1 subjects [48] are in agreement with the above findings of impaired low-level visual processing.

Another main result of the present study focused that the ability to integrate visual stimuli into a coherent structure to reproduce the complexity of the figure is impaired in NF1. The copy strategy is important to investigate *how* the subject approaches the task. A possible hypothesis to explain the poorer performance of NF1 in copy and short- and long-term recall could be that visual-perception impairment is part of a more general central coherence deficit, with a heightened focus on details rather than the whole picture and difficulties in the integration of separate features [49, 50].

The distribution of the copy strategy is significantly different among the three groups. The children with NF1 used a less efficient strategy of RCFT copy as compared to the control groups; in particular, 5 (28%) of them copied details on a confused ground with little structure in which the global model was poorly recognizable. It is a poor copy strategy which was not used by any S or TD subject. On the other hand, the majority of typical developing subjects used a more efficient strategy based on drawing the details of the armature, with a significantly higher frequency than the NF1 and siblings. The recent study by Van Eylen et al. [30] also found a more fragmented and detail-oriented processing style of RCFT in children with NF1 compared to TD children. A significant whole-sample correlation was found between a more fragmented processing style and increased inhibition cost on an executive functioning task, but the effect of the group remained significant when including executive measure as covariate, despite no significant differences were found between the NF1 group and the TD group.

In our sample, the copy strategies are positively correlated with both copy (derived from the sum of the analysis of the form and placement of the stimulus) and recall performances, confirming that a more configural/holistic style may facilitate both the visual-perceptual and visuomotor *ability* and the recall process. However, when controlling for the type of strategy as a covariate into the ANOVA model, the effect of the group on RCFT measures remained significant, despite only the contrast between NF1 and TD remains significant. This result seems to confirm the specific role of visual-perceptual processing impairment in NF1 compared to siblings, while in comparison to TD children it appears more important the influence of strategies.

Although the correlational nature of our analyses does not support a causal interpretation of the results, the significant positive association between copy abilities and the type of implemented strategy emphasizes that poor performances may be partially influenced by the use of less functional planning strategies in NF1. Executive functions, necessary and concurrent with strategic choices, are known to be impaired in children with NF1 regardless of the organization of visual stimuli [3]. A malfunctioning of executive functions in NF1 has been actually reported in planning tasks without external indications in which the subject has to rely entirely on self-organization and monitoring [51, 52]. A recent case-control study using the Behavioural Assessment of the Dysexecutive Syndrome in children found significantly lower performance in NF1 than typical peers in the Key Search subtest, a task without any a priori rule given by the examiner. NF1 children understood the request and followed the instructions, but they were less able to find a strategy autonomously and to produce an efficient search pattern transferable to other similar real life situations [52].

Taken together, these findings confirm a reduced top-down information integration during tasks requiring *self-generated specific planning* in NF1 compared to typical peers.

In the present study, performance in both short- and long-term recalls (at 3 and 30 minutes after the copy) is significantly reduced in NF1 as compared to controls. A few studies investigated different types of memory in subjects with NF1, and results are still not consistent. Most authors did not find significant impairment in visual memory in children with NF1 compared to healthy children and unaffected siblings [2, 22–24]. On the other hand, RCFT studies show poor performance in children with NF1 compared to siblings or normal peers in delay recall condition [20, 21, 26].

In the present study, NF1 performances in the recall trials are less efficient than in the control groups; however, we observe quite stable *z*-scores compared to the copy, suggesting a fairly good retention of visuospatial acquired information, despite the poor performance in the visual processing and encoding phase (i.e., copy trial). The recognition is within the average range and similar to the control groups. The two types of memory are different and rely on distinct mechanisms: recall is based on recollection mechanisms that provide the qualitative aspects of a stimulus, with almost no contribution to the familiarity process [53]; the recognition trial is based on the ability of recognize different elements (details) of the figure, more than the figure in its complexity, and gets benefit from the consolidation of the stimulus derived from the copy and the two recall trials. It has been widely demonstrated that recognition memory performance reflects two distinct memory processes, i.e., the *recollection* of the stimulus and a sense of *familiarity* with the features of the stimulus, a rapid process which does not allow the recall of the stimulus' quality [53–55].

The main limitation of our study is the small sample size (still comparable with the majority of the studies) leading to reduced statistical power; its strengths, as mentioned in the Introduction, include the strict inclusion criteria and the a priori control of some confounding factors, such as the presence of brain tumours, intellectual disability, and neurological conditions like epilepsy. Moreover, we employed RCFT to assess different domains of visual processing, without the need to administer different psychometric instruments. This design allowed the study to remain in a defined theoretical framework [3].

We did not assess the association between visual-perceptual abilities and executive functions, because this would have been beyond the purpose of the present study, but the ANOVA results discussed above support a partial influence of strategic planning and pave the way for future investigations. In our sample, 44% of NF1 patients score over the significance cut-off at attention problem scales, in line with previous experimental studies suggesting a high comorbidity of NF1 with ADHD [2]. However, no significant correlation has been found between ADHD indices and copy performances, disconfirming the association between copy processing style and ADHD symptoms as a possible confounding factor. Conversely, it is possible that the absence of correlation may be due to weak statistical power; thus, the role of attention deficit in visual-perception impairment in NF1 is a still open issue.

Finally, we did not include the IQ scores as a covariate in the ANOVA as suggested by Dennis et al. [56]. There is an ongoing debate about this issue in the literature on neurodevelopmental disorders [3]. In our opinion, using IQ as a covariate may produce overcorrected and anomalous findings; if there is shared variance between IQ and visual-perceptual abilities, using IQ as a covariate may reduce real group differences and thus bias results [56]. Van Eylen et al. also found that the co-occurring lower IQ of the children with NF1 did not impact substantially upon their visuoperceptual performance thus bounding the role of IQ as a confounding factor [30].

## 5. Conclusions

The present study provides new evidences about visuospatial impairment in NF1, studying several different functions in an accurately selected developmental sample. Deficits in perceptual analysis of shapes and their spatial features, in visuomotor integration efficiency, and in recall memory are confirmed as specific characteristics of the disorder, while the recognition memory seems to be preserved; further insight has been provided about the role of poorly efficient processing strategies.

In line with results of neuroimaging studies, we suggest that early visual processing may influence higher order functions such as planning and strategy choice [7], in addition to an impairment in executive function [3].

In the light of our results, memory disorders in NF1 patients seem to involve memory circuits related to *recollection*, but not to *familiarity*. Visual deficits are wide ranging and can be responsible for severe difficulties in daily life and in academic skills.

Finally, our data confirm the wide phenotypic cognitive and neuropsychological range, related to several processes underpinned by at least partially separated cerebral networks. This variability within and between subjects suggests that NF1 is composed of numerous distinct diseases, each defined by intricate influences, such as genetic, demographic, and microenvironmental factors [57].

## Figures and Tables

**Table 1:** Group comparison on the WISC-III Intelligence test.

	NF1 mean (SD)	S mean (SD)	TD mean (SD)	One-way ANOVA			
				F	*p* value^∗^	Post hoc tests^§^ NF1 < S	Post hoc tests NF1 < TD
*Wechsler scale*							
Full-scale IQ	98 (12)	112 (8)	111 (11)	9.649	≤0.001	*p* = 0.01	*p* = 0.001
VC	98 (12)	105 (14)	106 (12)	2.463	0.96	n.s	n.s
PO	101 (14)	116 (7)	113 (11)	8.679	0.001	*p* = 0.001	*p* = 0.006
FFD	94 (12)	108 (6)	113 (11)	15.316	≤0.001	*p* = 0.02	*p* ≤ 0.001
PS	98 (14)	104 (12)	107 (15)	2.136	0.129	n.s	n.s

Legend. NF1: neurofibromatosis type 1; S: siblings; TD: typically developing children; VC: verbal comprehension; OP: perceptual organization; FFD: freedom from distractibility; PS: processing speed. ^∗^ *p* values of 0.05 or lower are considered significant. ^§^Post hoc Bonferroni correction.

**Table 2:** Description and comparisons of the group performance on the RCFT.

	NF1 mean (SD)	S mean (SD)	TD mean (SD)	One-way ANOVA				One-way ANOVA controlling for strategy			
				*F*	*p* value^∗^	Post hoc tests^§^ NF1 < S	Post hoc tests NF1 < TD	*F*	*p* value	Post hoc tests NF1 < S	Post hoc tests NF1 < TD
*RCFT (z-score)*											
Copy	-1.90 (1.89)	-0.32 (0.95)	-0.38 (1.41)	6.563	0.003	*p* = 0.008	*p* = 0.010	3.176	0.050	*p* = 0.048	*p* = 0.306
Immediate recall	-1.53 (0.96)	-0.41 (1.10)	-0.28 (1.06)	7.848	0.001	*p* = 0.007	*p* = 0.002	3.869	0.028	*p* = 0.040	*p* = 0.078
Delay recall	-1.63 (0.88)	-0.35 (1.10)	-0.37 (0.81)	8.619	0.001	*p* = 0.001	*p* = 0.005	4.975	0.011	*p* = 0.009	*p* = 0.169
Recognition	-0.18 (0.96)	-0.31 (1.10)	0.05 (1.45)	0.402	0.671	n.s.	n.s.	0.388	0.680	n.s.	n.s.

Legend. NF1: neurofibromatosis type 1; S: siblings; TD: typically developing children; RCFT: Rey Complex Figure Test. ^∗^ *p* values of 0.05 or lower are considered significant. ^§^Post hoc Bonferroni correction.

**Table 3:** Group comparison on the RCFT accuracy and placement of the copy.

	NF1 mean (SD)	S mean (SD)	TD mean (SD)	*F*	*p* value^∗^	Post hoc test^§^
Accuracy	12.8 (3.77)	15.35 (3.92)	14.92 (2.22)	2.815	0.069	NF1 < S (*p* = 0.096) NF1 < TD (*p* = 0.211)
Placement	14.06 (3.84)	16.18 (3.78)	16.83 (1.49)	3.658	0.033	NF1 < S (*p* = 0.17) NF1 < TD (*p* = 0.037)

Legend. NF1: neurofibromatosis type 1; S: siblings; TD: typically developing children. ^∗^ *p* values of 0.05 or lower are considered significant. ^§^Post hoc Bonferroni correction.

**Table 4:** RCFT performance distributions within each of the three groups.

RCFT measure	Performance levels^§^	NF1 *N* (%) Residual^∗^	S *N* (%) Residual	TD *N* (%) Residual	*χ* ^2^	Exact *p*
Copy	>-1 SD	6 (33.3) **-2.9**	13 (76.5) 1.6	13 (72.2) 1.3	9.749	0.038
	<-1 SD	4 (22.2) 0.7	3 (17.6) 0.1	2 (11.1) -0.8		
	<-2 SD	8 (44.4) **2.7**	1 (5.9) **-2.0**	3 (16.7) -0.7		

Immediate recall	>-1 SD	5 (27.8) **-3.3**	11 (64.7) 0.6	15 (83.3) **2.6**	13.517	0.005
	<-1 SD	6 (33.3) 1.1	5 (29.4) 0.6	2 (11.1) -1.6		
	<-2 SD	7 (38.9) **3.0**	1 (5.9) -1.6	1 (5.6) -1.6		

Delay Recall	>-1 SD	3 (16.7) **-3.6**	11 (64.7) 1.4	13 (72.2) **2.2**	17.924	0.001
	<-1 SD	7 (38.9) 1	6 (35.3) 0.6	3 (16.7) -1.5		
	<-2 SD	8 (44.4) **3.4**	0 (0.0) **-2.4**	2 (11.1) -1.0		

Recognition	>-1 SD	15 (83.3) 0.7	11 (64.7) -1.5	15 (83.3) 0.7	6.901	0.071
	<-1 SD	3 (16.7) -0.3	6 (35.3) **2.1**	1 (5.6) -1.8		
	<-2 SD	0 (0.0) -1.0	0 (0) -1.0	2 (11.1) 2.0		

Legend. NF1: neurofibromatosis type 1; S: siblings; TD: typically developing children; RCFT: Rey Complex Figure Test. ^∗^The significant corrected standardized residuals are bolded. ^§^Performance levels are represented as follows: >-1 SD: normal, <-1 SD: borderline, <-2 SD: impaired.

**Table 5:** Visual-processing style distribution among the three groups.

Strategy	NF1 *N* Residual^∗^	S *N* Residual	TD *N* Residual	Total
Detection of the armature	0	3	2	5
	-1.7	1.4	0.3	
Details of armature	6	6	13	25
	-1.4	-1.2	**2.6**	
Juxtaposition of details	7	7	3	17
	0.8	1.0	-1.7	
Details on a confused ground	5	1	0	6
	**2.7**	-0.9	-1.9	

Legend. NF1: neurofibromatosis type 1; S: siblings; TD: typically developing children. ^∗^The significant corrected standardized residuals are bolded.

## Data Availability

The neuropsychological data used to support the findings of this study are available from the corresponding author upon request.

## Supplementary Materials

Supplementary Materials Detailed Rey Complex Figure Test Administration.
