# Supplementary material for: Visuoperceptual Impairment in Children with NF1: From Early Visual Processing to Procedural Strategies
Source: Behav Neurol. 2019 Jan 13;2019:7146168. doi: 10.1155/2019/7146168 (PMC6348799; doi:10.1155/2019/7146168)
Supplement: Supplementary Materials — Detailed Rey Complex Figure Test Administration. [file 7146168.f1.pdf]

Supplementary Materials

Rey Complex Figure Text Administration

Administration of the RCFT involves a Copy trial, a 3-minute Immediate Recall trial, 30-minute Delayed Recall trial and a newly developed Recognition trial [1] which is administered immediately after the Delayed Recall trial. The recognition trial presents 12 of the 18 scoring elements of the complex figure stimulus, along with 12 designs that serve as foils. The respondent indicates which items are recognized from the earlier Copy trial. In addition to the newly developed Recognition trial, this version of the complex Rey Figure also presents empirically derived scoring criteria that improve the clarity and objectivity of the 36 point scoring system. Specifically more precise rules have been developed to resolve ambiguities in scoring the accuracy and placement of each of the 18 scoring units. Normative data are provided for children and adolescents 6 through 17 years (Supplement norms for children and adolescents [2]) and for adult 18 through 89 years of age [3].

A score of 0, 0,5, 1 or 2 is assigned to each units of the figure based on accuracy and placement criteria. Unit score are then summed to obtain the raw score for that drawing and the same scoring criteria apply to all three drawing trials (Copy, Immediate Recall, Deleyed Recall).

The Recognition score is the sum of Recognition True Positive (i.e. the number of elements that are correctly identified as part of the figure stimulus) and Recognition True Negatives (i.e. the number of elements that are correctly identified as not belonging to the figure stimulus).

References

[1] Meyers, J., & Lange, D. (1994). A Recognition Subtest for the Complex Figure. Clinical Neuropsychologist 8:153–166.

[2] Meyers, J.E., & Meyers, K.R. (1996). *Rey Complex Figure Test and recognition trial: Professional manual. Supplemental norms for children and adolescents.* Psychological Assessment Resources.

[3] Meyers JE, & Meyers KR. (1995). *Rey Complex Figure Test and recognition trial professional manual.* Psychological Assessment Resources.
